# Molecular Dissection of the Primase and Polymerase Activities of Deep-Sea Phage NrS-1 Primase-Polymerase

**DOI:** 10.3389/fmicb.2021.766612

**Published:** 2021-12-17

**Authors:** Fengtao Huang, Xueling Lu, Chunxiao Yu, Piotr Sliz, Longfei Wang, Bin Zhu

**Affiliations:** ^1^Key Laboratory of Molecular Biophysics, The Ministry of Education, College of Life Science and Technology and Shenzhen College, Huazhong University of Science and Technology, Wuhan, China; ^2^Department of Biological Chemistry and Molecular Pharmacology, Harvard Medical School, Boston, MA, United States; ^3^Program in Cellular and Molecular Medicine, Boston Children’s Hospital, Boston, MA, United States; ^4^School of Pharmaceutical Sciences, Wuhan University, Wuhan, China

**Keywords:** DNA polymerase, primase, DNA replication, *de novo* synthesis, phage

## Abstract

PrimPols are a class of primases that belong to the archaeo-eukaryotic primase (AEP) superfamily but have both primase and DNA polymerase activities. Replicative polymerase from NrS-1 phage (NrSPol) is a representative of the PrimPols. In this study, we identified key residues for the catalytic activity of NrSPol and found that a loop in NrSPol functionally replaces the zinc finger motif that is commonly found in other AEP family proteins. A helix bundle domain (HBD), conserved in the AEP superfamily, was recently reported to bind to the primase recognition site and to be crucial for initiation of primer synthesis. We found that NrSPol can recognize different primase recognition sites, and that the initiation site for primer synthesis is not stringent, suggesting that the HBD conformation is flexible. More importantly, we found that although the HBD-inactivating mutation impairs the primase activity of NrSPol, it significantly enhances the DNA polymerase activity, indicating that the HBD hinders the DNA polymerase activity. The conflict between the primase activity and the DNA polymerase activity in a single protein with the same catalytic domain may be one reason for why DNA polymerases are generally unable to synthesize DNA *de novo*.

## Introduction

PrimPols are a class of primases possessing both the primase and DNA polymerase activities. The enzymes are first identified from archaeal plasmid pRN1 (Lipps et al., 2003) and has subsequently been found in various organisms including bacteria, bacteriophages, and humans (Halgasova et al., 2012; Sanchez-Berrondo et al., 2012; García-Gómez et al., 2013; Wan et al., 2013; Picher et al., 2016; Zhu et al., 2017; Gupta et al., 2019). Based on their structures and sequences, PrimPols are grouped into the archaeo-eukaryotic primase (AEP) superfamily (Iyer et al., 2005; Guilliam et al., 2015; Kazlauskas et al., 2018). However, unlike conventional AEP members that function as replication initiation-specific primases, PrimPols play diverse roles in different organisms (Guilliam et al., 2015). For example, PrimPol from archaeal plasmid pRN1 (ORF904) is suggested to be a replicative DNA polymerase (Berkner et al., 2014), while human PrimPol (hPrimPol) is involved in DNA damage tolerance (Bianchi et al., 2013; García-Gómez et al., 2013). Recently, it is reported that a PrimPol encoded in a mobile element of *Thermus thermophilus* is contributed in defense against invading DNA (García-Quintans et al., 2020). To date, the most extensively studied PrimPols are ORF904 and hPrimPol. ORF904 contains an N-terminal PrimPol catalytic domain and a C-terminal superfamily 3 helicase domain (Lipps et al., 2003; Sanchez et al., 2009). The PrimPol domain of ORF904 recognizes a 5'-GTG-3' motif in the template DNA and synthesizes about 8 nt mixed primer composed of a single ribonucleotide at the 5' end following deoxynucleotides (Beck and Lipps, 2007). It was suggested that ORF904 initiates primer synthesis outside of the 5'-GTG-3' motif, while hPrimPol and some microbial PrimPols (BcMCM and TthPrimPol) can recognize a 5'-CCTG-3' motif and initiate the primer synthesis inside of the 5'-CCTG-3' motif (Sanchez-Berrondo et al., 2012; García-Gómez et al., 2013; Picher et al., 2016). There is a helix bundle domain (HBD) at the C-terminus of the primase part of ORF904, which is critical for primase activity but not for primer extension activity (Beck et al., 2010). For example, the point mutations W314A and Y352A in the HBD of ORF904 significantly reduce the primase activity (Beck et al., 2010). Recently, it was found that ATP binding induces conformational changes of the HBD of ORF904 that allow it to specifically recognize the 5'-GTG-3' motif and prepare primer synthesis, demonstrating the critical role of the HBD in DNA template recognition and initiation of primer synthesis (Boudet et al., 2019). As the HBD is conserved in the AEP superfamily, it is likely to play a general role in all AEP superfamily proteins (Boudet et al., 2019).

The DNA polymerase from deep-sea phage NrS-1 (NrSPol) also belongs to the PrimPol family (Zhu et al., 2017). The domain organization of NrSPol is similar to that of ORF904, while the PrimPol domain of NrSPol lacks the typical zinc finger motif found in ORF904 and many other AEP family proteins, and the C-terminus of NrSPol has a unique helicase domain that forms a ring-shaped architecture and increases the polymerization activity of NrSPol (Zhu et al., 2017; Chen et al., 2020). Moreover, unlike ORF904, NrSPol recognizes a specific sequence motif (5'-TTTGGTTA-3') and initiates DNA synthesis exclusively using dNTPs (Zhu et al., 2017). The processivity of NrSPol is dramatically enhanced when associated with NrS-1 phage-encoded helicase and ssDNA-binding protein, indicating that NrSPol is a replicative DNA polymerase capable of synthesizing the NrS-1 phage genome *de novo* (Zhu et al., 2017).

Owing to special origin and potential physiological role, NrSPol is an ideal research model for understanding the mechanism and evolution of PrimPols. In this study, we systematically examined the effects of potentially important amino acid residues on the activities of NrSPol. We found several amino acid residues that are critical for the primase activity and primer extension activity. Unexpectedly, we found that mutations in the HBD inactivate primase activity but enhance DNA polymerase activity. We also observed flexibility in the template recognition and initiation of the primase activity of NrSPol. The results have expanded our understanding of the catalytic activities of NrSPol and other PrimPol family proteins.

## Materials and Methods

### Plasmid Construction

The plasmids for the expression of recombinant NrSPol (pET28b-NrSPol) and the N-terminal 300 amino acid residues (N300) of NrSPol were constructed in previous work (Zhu et al., 2017). The sequence encoding the TEV protease cleavage site (ENLYFQ/G) was inserted upstream of N300 coding DNA to generate plasmid pET28b-His-TEV-N300 to facilitate 6 × His-tag removal. Mutants of the full-length NrSPol and N300 were constructed by whole-plasmid PCR.

### Expression and Purification of Recombinant Proteins for Enzymatic Assays

Recombinant NrSPol was prepared as previously described with slight modification (Zhu et al., 2017). *Escherichia coli* BL21(DE3) cells harboring the expression plasmid pET28b-NrSPol were grown in LB medium containing 50 μg/ml kanamycin at 37°C until the optical density at 600 nm reached about 1.0. Protein expression was induced by the addition of IPTG to a final concentration of 0.5 mM at 25°C followed by incubation for another 12 h. The cells were harvested and lysed by sonication, and the N-terminal His-tagged NrSPol was purified using a Ni^2+^-chelating affinity column. Purified protein was dialyzed against storage buffer (50 mM Tris-HCl, pH 7.5, 0.1 mM DTT, 0.1 mM EDTA, and 50% glycerol) and then stored at −20°C. Recombinant mutant proteins were purified using a similar procedure as described above. The purified proteins were analyzed by SDS-PAGE (Supplementary Figure S1).

### Purification of N300 for Crystallization

N300 was expressed in *E. coli* LOBSTER strain and was purified using a Ni^2+^-chelating affinity column. 6 × His-tag was removed by TEV cleavage overnight at 4°C. The N300 was further purified by FPLC using a heparin column and a size exclusion column (GE Healthcare). The final storage buffer for N300 contained 20 mM Tris-HCl (pH 8.0), 100 mM sodium chloride, 1 mM magnesium chloride, and 5 mM β-mercaptoethanol (BME).

The selenomethionine (SeMet) substituted N300 sample was expressed using a previously described method (Van Duyne et al., 1993) and then purified using the same procedure. The final storage buffer for N300 contained 20 mM Tris-HCl (pH 8.0), 100 mM sodium chloride, and 5 mM BME.

### Crystallization of N300

The SeMet N300 sample was concentrated to 10 mg/ml and then incubated with ddCTP in a 1:1.2 ratio. The N300 sample was concentrated to 10 mg/ml and then incubated with ddCTP and ssDNA in a 1:1.2:1 ratio. The N300 samples were then mixed with crystallization buffer in a 1:1 ratio and crystallized overnight using sitting drop vapor diffusion: native N300 was crystalized in 800 mM sodium phosphate monobasic, 1,200 mM potassium phosphate dibasic, and 100 mM sodium acetate (pH 4.5); SeMet N300 was crystallized in 20% (w/v) PEG 3000, 200 mM calcium acetate, and 100 mM Tris-HCl (pH 7.0).

### Data Collection

The native and SeMet N300 crystals were frozen in the mother liquor by the addition of 10% glycerol. The diffraction images were collected using x-rays with a wavelength of 0.9799 at APS (Advanced Photon Source, Chicago). The native N300 crystals diffract to 1.86 Å in space group C2 and SeMet N300 crystals diffract to 2.24 Å in space group P2_1_2_1_2_1_.

### Structure Determination

The diffraction images of native and SeMet N300 were indexed, integrated, and merged using xia2 (Winter et al., 2013). Statistics of the merged datasets are shown in Supplementary Table S1. The SAD method was used to phase the SeMet N300 crystals and the initial model containing about 100 amino acid residues was built using phenix.autosol (Adams et al., 2010; Winter et al., 2013). The remainder of the model was manually built based on the electron density using coot (Emsley and Cowtan, 2004). The structure of native N300 was solved by molecular replacement using phaser (McCoy et al., 2007). Both native and SeMet N300 structural models were refined by iterating between auto refinement (phenix.refine) and manual adjustment in coot. The statistics of the final models are shown in Supplementary Table S1.

### Primer Extension Assays

A 16-nt primer (5'-CATGTCAGGGTCTTCA-3') was labeled at 5'-end using T4 polynucleotide kinase (New England Biolabs) and [γ-^32^P] ATP (PerkinElmer), and then annealed to a 36-nt template (5'-GAGATCCTATCGAGTAGCTCTGAAGACCCTGACATG-3'). The primer extension reactions were performed as described previously (Zhu et al., 2017). Unless otherwise stated, the reaction mixtures (10 μl) containing 20 mM Tris-HCl (pH 8.8), 10 mM (NH_4_)_2_SO_4_, 10 mM KCl, 0.1% Triton X-100, 5 mM MgSO_4_, 0.5 mM dNTPs, 50 nM 5'-labeled primer/template duplex, and 0.5 μM NrSPol, N300, or their mutants were incubated at 50°C for indicated time. Reactions were terminated by adding 5 μl of 95% formamide dye containing 15 mM EDTA. Samples were heated for 3 min at 90°C and analyzed by 10% denaturing PAGE containing 7 M urea. The primer extension activities of NrSPol, N300, and their mutants were also evaluated using primed M13 ssDNA (annealed with 5'-^32^P-labeled M13 primer M2, 5'-CCCAGTCACGACGTT-3') as the substrate. Reactions were performed similarly to the primer/template duplex extension assays, except for the DNA templates used. Samples were analyzed by 2% alkaline agarose electrophoresis. A 2-Log DNA ladder (New England Biolabs) as a size marker was labeled at the 5'-end using T4 polynucleotide kinase and [γ-^32^P] ATP.

### Primase Assays

Unless otherwise stated, the reaction mixtures (10 μl) contained 20 mM Tris-HCl (pH 8.8), 10 mM (NH_4_)_2_SO_4_, 10 mM KCl, 0.1% Triton X-100, 5 mM MgSO_4_, 50 μM of the indicated types of dNTPs, trace amounts of [α-^32^P] dATP (PerkinElmer), 10 μM oligodeoxynucleotide template or 5 nM M13 ssDNA (New England Biolabs), and 0.5 μM NrSPol, N300, or their mutants. Reactions were incubated for 30 min at 50°C, and then were stopped by adding 5 μl of 95% formamide dye containing 15 mM EDTA. Samples were boiled for 3 min at 90°C and analyzed by 25% PAGE containing 3 M urea. The RNA primer synthesis reactions were as for the DNA primer synthesis reaction, except that NTPs and [α-^32^P] ATP (PerkinElmer) replaced dNTPs and [α-^32^P] dATP in the reaction mixture. After quenching the reaction, the samples were heated for 3 min at 75°C and analyzed by 25% PAGE containing 3 M urea.

### *De novo* DNA Synthesis on M13 ssDNA Template

The reaction mixtures (10 μl) contained 20 mM Tris-HCl (pH 8.8), 10 mM (NH_4_)_2_SO_4_, 10 mM KCl, 0.1% Triton X-100, 5 mM MgSO_4_, 50 μM dNTPs, trace amounts of [α-^32^P] dATP, indicated concentrations of M13 ssDNA, and indicated concentrations of NrSPol, N300, or their mutants. Reactions were incubated for 30 min at 50°C and then quenched by adding 2 μl alkaline loading buffer (300 mM NaOH, 6 mM EDTA, 18% Ficoll 400, 0.15% bromocresol green, and 0.25% xylene cyanol FF). Samples were analyzed by alkaline agarose electrophoresis. Lambda DNA ladder as a size marker was labeled at the 3'-end using Klenow Fragment (New England Biolabs) and [α-^32^P] dATP.

## Results

### Crystal Structure of N300 Shows a Serine-Rich Loop in the Active Site

**Figure 1:**
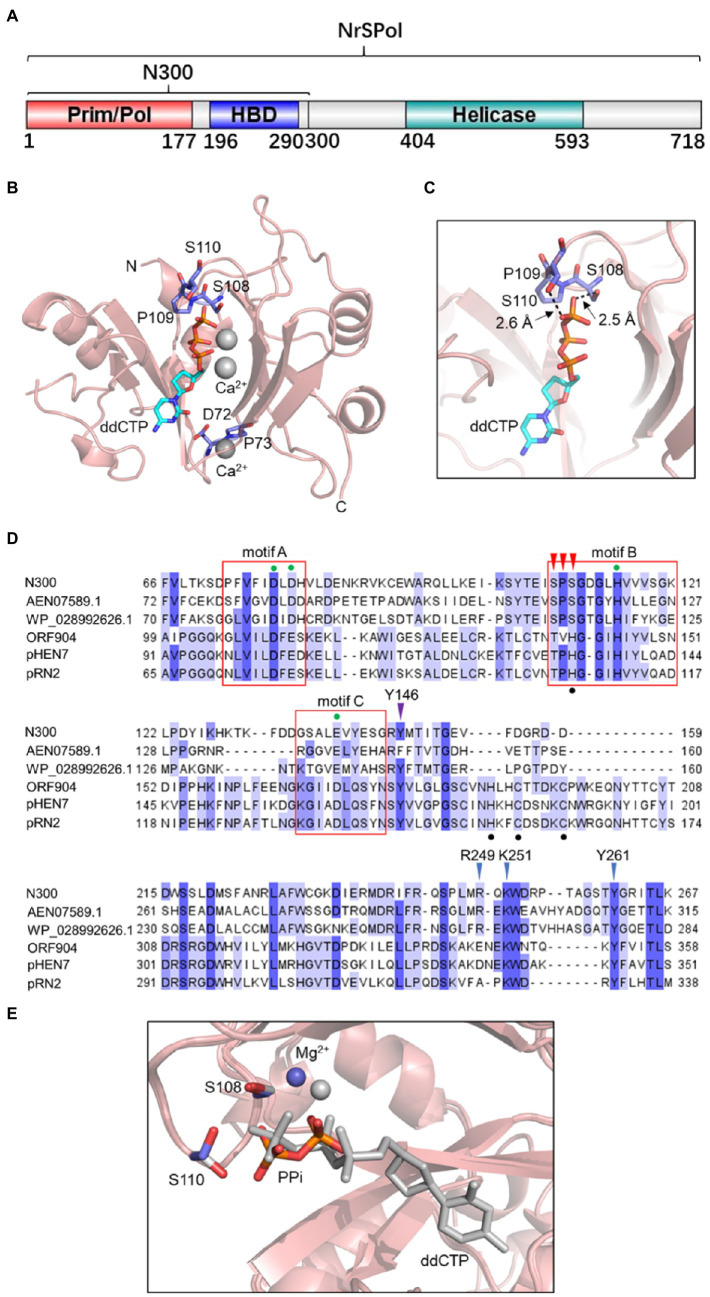
A serine-rich (SPS) loop in the N300 structure. **(A)** The domain architecture of NrS-1 phage polymerase (NrSPol). **(B)** Ribbon diagram of N300. The Prim/Pol domain adopts a saddle-shaped fold. The ddCTP and two Ca^2+^ ions are located at the central cleft and are coordinated by a serine-rich (SPS) loop. A chelated metal ion is found at the bottom of N300 structure and is assigned to Ca^2+^ due to the high Ca^2+^ concentration in the crystal conditions. **(C)** Detailed interactions between S108, S110, and the γ-phosphate of ddCTP. **(D)** Sequence alignment of the catalytic domain of N300 and other PrimPol proteins. The red boxes indicate the motif A, B, and C. The green dots indicate the key residues in the motif A, B, and C. The black dots indicate the zinc finger motif present in ORF904 and its orthologs. The red arrowheads indicate the SPS motif in N300 and its orthologs. The purple arrowhead indicates the position of Y146 of N300. The blue arrowheads indicate the positions of key residues R249, K251, Y261 in the HBD domain of N300. More details for the alignment of archaeo-eukaryotic primase (AEP) family proteins including BcMCM and TthPrimPol can be seen elsewhere (Picher et al., 2016). **(E)** Overlay of PPi/Mg^2+^-bound N300 structure (colored) and ddCTP/Ca^2+^-bound N300 structure (gray).

In our previous study, N300 exhibited similar primase and DNA polymerase activities to those of the full-length enzyme (Zhu et al., 2017). In this study, to explore the structural basis of N300’s primase and primer extension functions, we determined the crystal structures of N300 in complex with ddCTP. The structure of N300 consists of two domains: a primase-like (Prim/Pol) domain located at the N-termini and a HBD at the C-termini (Figures 1A,B). The two domains are connected by a linker region (178–195), of which electron density is missing due to flexibility. As is shown in Figure 1B, the primase-like domain adopts a saddle-shaped fold that resembles the previously reported PrimPol domain of ORF904 (Lipps et al., 2004) but with several significant differences. Frist, the primase-like domain of NrSPol utilizes a serine-rich loop with an SPS motif (S108, P109, and S110) instead of the very conserved zinc finger motif in the active site (Figures 1B,C,D). Interestingly, the electron density of the S108 side chain extends to the γ-phosphate of ddCTP with a close O-O distance of 2.5 Å, suggesting a strong hydrogen bond (Figure 1C). The side chain of S110 is also adjacent to the γ phosphate of ddCTP, with a O-O distance over 2.6 Å (Figure 1C). This structure indicates that S108 and S110 coordinate ddCTP and are likely critical for N300’s primase and polymerase activities. Second, a chelated metal ion is found at the bottom of the N300 structure that is not present in any other PrimPol or primase structures (Figure 1B). Given the high Ca^2+^ concentration in the crystal solution (100 mM), this metal ion is assigned as Ca^2+^, which is coordinated by the main chain carbonyl oxygens of T69, D72, F74, and E142.

We next attempted to capture the structure of N300 in action and crystalized N300 with ddCTP and Mg^2+^. Although, the space group for this crystal form is different from N300/ddCTP/Ca^2+^, we observed that a pyrophosphate (PPi) occupies the active site instead of ddCTP (Figure 1E). Since PPi is not present during protein purification and crystallization, we reasoned that the observed PPi is the product of ddCTP hydrolysis. Interestingly, the PPi adopts a different conformation compared to ddCTP and moves further from S108 and S110, which also relocates the metal ion (Mg^2+^ instead of Ca^2+^ in this structure) by 1.4 Å (Figure 1E). As a result, the side chain of S110 is rotated by about 120°. This conformation change of the PPi likely facilitates the release of the PPi after dNTP hydrolysis.

### Key Amino Acid Residues That Are Involved in dNTP Binding, Affect the Primase and Primer Extension Activities of NrSPol

**Figure 2:**
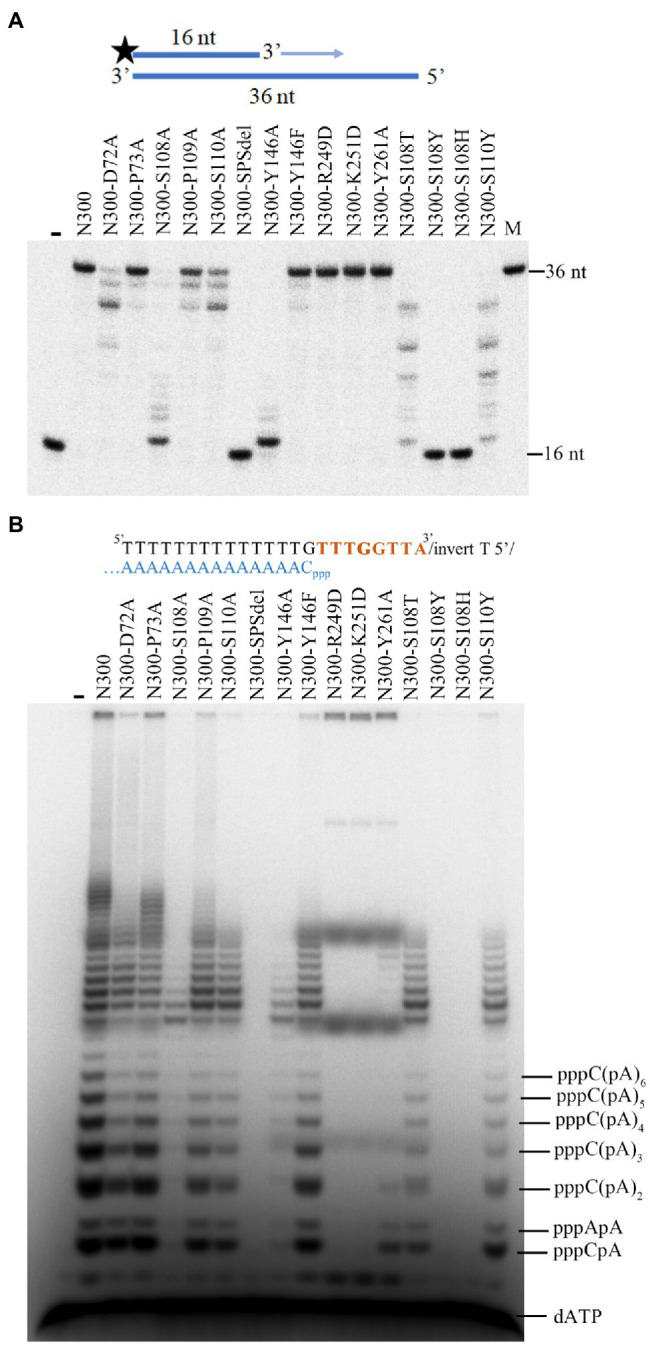
The DNA polymerase and primase activities of N300 and its mutants. **(A)** Extension of ^32^P-labeled 16/36-mer primer/template duplex substrate by N300 and its mutants. The reactions were incubated for 20 min at 50°C, and the products were separated on a 7 M urea-10% PAGE. **(B)** Primer synthesis on a designed 23-nt DNA template by N300 and its mutants as described previously (Zhu et al., 2017). The reactions were incubated for 30 min at 50°C, and the products were separated on a 3 M urea-25% PAGE. Data in the figure are representative of at least two independent experiments.

Based on the structure of N300, we constructed various mutants of N300 to examine the roles of possible key residues. D72 is likely to coordinate with the additional metal ion (Figure 1B). To test its role, the residue was substituted with Ala. In addition, the adjacent residue P73 was also substituted with Ala as a control. Compared to N300 and P73A mutation, the D72A mutation only slightly decreases primer extension activity and primase activity of N300 (Figures 2A,B), indicating that D72 is not a key residue for metal ion coordination.

The zinc finger motif is found in many AEP family proteins, such as ORF904, but not in NrSPol. As shown in Figure 1C, a loop that consists of residues S108, P109, and S110 in the Prim/Pol domain of NrSPol seems to functionally replace the zinc finger motif that is considered to bind the incoming dNTP (Lipps et al., 2004). To investigate the role of the loop for the catalytic activity of N300, single-residue mutants of the loop of N300 and a mutant with the deletion of the three residues (N300-SPSdel) were constructed, and their activities were tested. The S108A mutation in N300 dramatically reduces both primer extension and primase activities; the S110A mutation also reduces the catalytic activity to a lesser extent, and P109A mutation slightly affects the catalytic activity (Figures 2A,B). S110Y mutation also decreases the catalytic activity to some extent, while S108Y mutation, S108H mutation, or deletion of the loop completely abolishes the catalytic activity of N300 (Figures 2A,B), further indicating that S108 is a key residue for the catalytic activity of N300. The side chain of T108 residue is similar to that of S108, thus S108T mutation shows some catalytic activity. However, the side chain of Y108 or H108 residue is very different from the side chain of S108. S108Y and S108H mutations may not only directly affect nucleotide binding but also affect the orientation of key residues G111 and G113. Thus, S108Y and S108H mutations completely abolish the catalytic activity. In addition, the overall shape of the circular-dichroism spectrum of N300-SPSdel is essentially identical to that of the N300 (Supplementary Figure S2), indicating that deletion of the SPS loop did not result in a significant structural perturbation. Collectively, SPS loop is involved in 3'-site dNTP interaction, especially the S108 residue.

Previous studies have shown that the HBD of ORF904 affects its primase activity but not the primer extension activity (Beck et al., 2010; Boudet et al., 2019). In this study, effects of the HBD mutations (R249D, K251D, and Y261A) of N300 on the primase activity and primer extension activity were investigated (Figure 1D). As shown in Figures 2A,B, mutants N300-R249D, N300-K251D, and N300-Y261A show much weaker primase activity than that of N300 but have comparable primer extension activity. The results are consistent with previous findings (Beck et al., 2010; Boudet et al., 2019).

### HBD-Inactivating Mutations Improve the DNA Polymerase Activity

**Figure 3:**
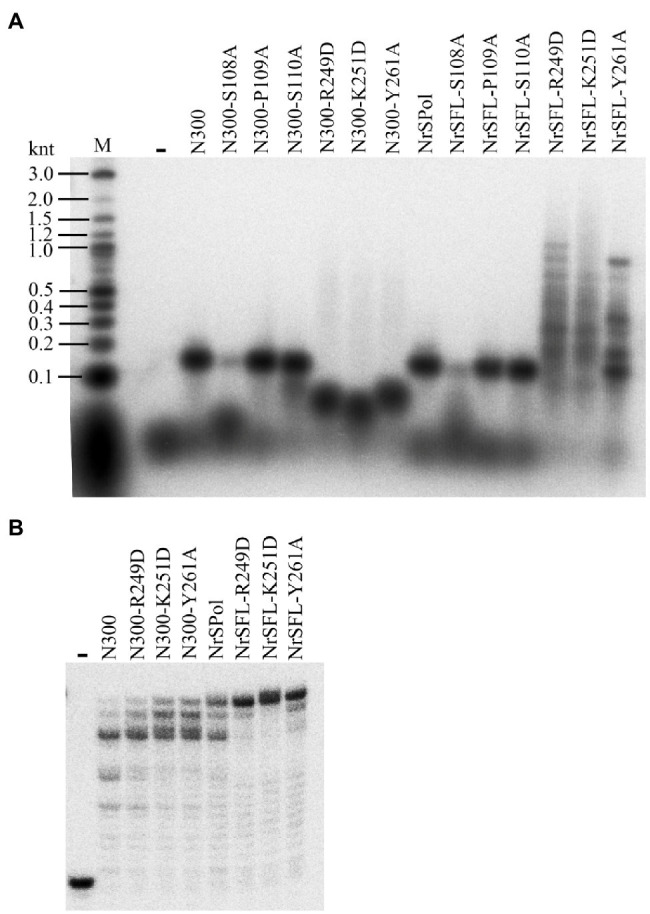
Enhanced primer extension activities of mutants of full-length NrSPol. **(A)** Primer extension assays with 5 nM primed-M13 ssDNA (annealed with ^32^P-labeled M13 primer, 5'-CCCAGTCACGACGTT-3') substrate. The reactions were incubated for 30 min at 50°C and the products were separated on a 2% alkaline agarose gel. Data in the Figure **A** are representative of two independent experiments. **(B)** Primer extension assays with ^32^P-labeled 16/36-mer primer/template duplex substrate. The reactions were incubated for 6 min at 50°C, and the products were separated on a 7 M urea-10% PAGE.

To further investigate the roles of some key residues in primer extension activity, the corresponding mutants of full-length NrSPol (NrSFL-S108A, NrSFL-P109A, NrSFL-S110A, NrSFL-R249D, NrSFL-K251D, and NrSFL-Y261A) were constructed, and the primer extension activities of mutants of both NrSPol and N300 were assayed on the M13 ssDNA template. Consistent with the results shown in Figure 2A, the primers are only slightly extended by N300-S108A or NrSFL-S108A on the M13 ssDNA template (Figure 3A). However, the results from HBD-inactivated mutants (N300-R249D, N300-K251D, N300-Y261A, NrSFL-R249D, NrSFL-K251D, and NrSFL-Y261A) are unexpected. The lengths of the major products synthesized by N300-R249D, N300-K251D, and N300-Y261A are much shorter than those synthesized by N300, while these mutants also synthesize trace amounts of products larger than those synthesized by N300 (Figure 3A). However, the lengths of the major products synthesized by NrSFL-R249D, NrSFL-K251D, and NrSFL-Y261A are much longer than those synthesized by NrSPol. These results suggest that R249D, K251D, or Y261A substitution in N300 impairs N300 processivity; conversely, R249D, K251D, or Y261A substitution in full-length NrSPol improves the processivity of the enzyme. Because the primer extension activities of N300, NrSPol and their HBD-inactivated mutants (R249D, K251D, or Y261A) show no difference on the 36-nt template when the reaction time was more than 20 min (Figure 2A), we further measured their activities with shorter reaction time of only 6 min. R249D, K251D, or Y261A substitutions in both N300 and NrSPol improve their primer extension activities, especially in NrSPol (Figure 3B). These results suggested that although HBD-inactivating mutations impair the primase activity, they can significantly enhance the DNA polymerase activity.

### HBD-Inactivating Mutations Enhance the Polymerase Processivity and Strand-Displacement Activity

**Figure 4:**
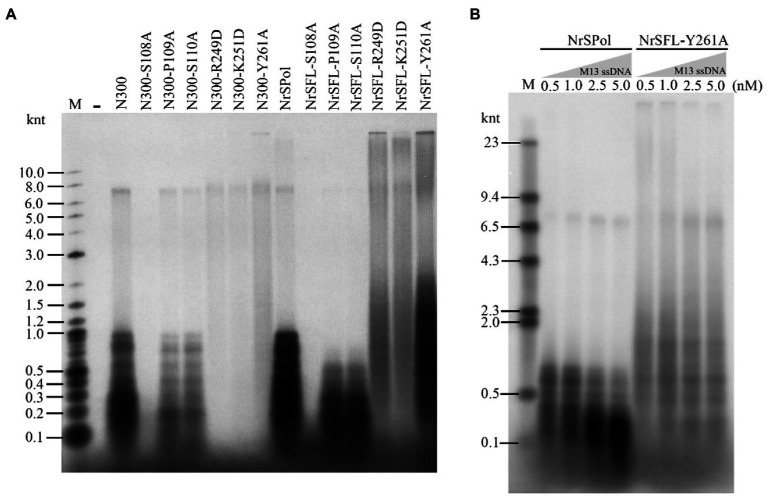
*de novo* DNA synthesis on the M13 ssDNA template by NrSPol, N300, and their mutants. **(A)** Reactions contained 1 nM M13 ssDNA and 500 nM NrSPol, N300, or their mutants, and were incubated for 30 min at 50°C. The products were separated on a 1% alkaline agarose gel. **(B)** Reactions contained 200 nM NrSPol or NrSFL-Y261A and various concentrations of M13 ssDNA and were incubated for 30 min at 50°C. The products were separated on a 0.6% alkaline agarose gel. Data in the figure are representative of at least two independent experiments.

We next sought to investigate the impact of these key residue substitutions on *de novo* DNA synthesis on the M13 ssDNA template. As expected, S108A mutations in N300 and NrSPol nearly abolish their activities for *de novo* DNA synthesis, and P109A or S110 mutations in N300 and NrSPol significantly decrease their activities for *de novo* DNA synthesis (Figure 4A). The HBD-inactivated mutants of N300 (N300-R249D, N300-K251D, and N300-Y261A) show very weak activities for *de novo* DNA synthesis (Figure 4A). The synthesis activities of HBD-inactivated mutants of NrSPol (NrSFL-R249D, NrSFL-K251D, and NrSFL-Y261A) are weaker than that of NrSPol, but their activities are much stronger than those of HBD-inactivated mutants of N300, especially NrSFL-Y261A that retains a relatively strong activity (Figure 4A). Notably, HBD-inactivated mutants of NrSPol also show a much higher processivity than that of NrSPol, and they can synthesize DNA longer than 10 knt, indicating that the HBD-inactivated mutants have relatively strong strand-displacement activities (Figure 4A). We next sought to investigate the processivity of NrSPol and NrSFL-Y261A in the presence of different concentrations of M13 ssDNA template. As shown in Figure 4B, the lengths of the products synthesized by NrSFL-Y261A are much longer than those synthesized by NrSPol, while the lengths of the products synthesized by both NrSPol and NrSFL-261A decrease with increasing M13 ssDNA concentration. In addition, the maximum length of the DNA products synthesized by NrSFL-Y261A is greater than 23 knt, which is much longer than the length of M13 ssDNA (7,249 nt), further demonstrating the strand-displacement DNA synthesis by NrSFL-Y261A (Figure 4B).

### Flexibility in the Primase Recognition Sequence

**Figure 5:**
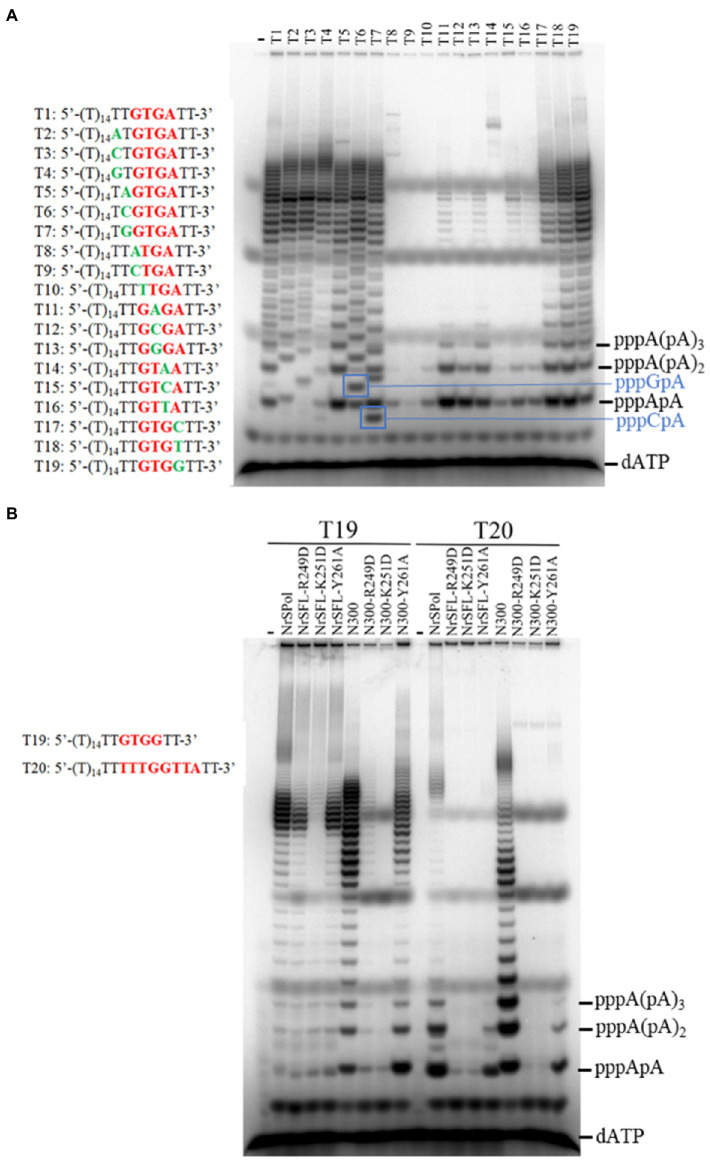
Identification of an efficient primase recognition site. **(A)** Dissection of the recognition sequence. Reactions contained 10 μM various oligodeoxynucleotide templates, 50 μM dNTPs, trace amounts of [α-^32^P] dATP, and 500 nM N300 and were incubated for 30 min at 50°C. **(B)** Comparison of the primer synthesis activity of NrSPol, N300, and their mutants on templates containing recognition sequences 5'-GTGG-3' and 5'-TTTGGTTA-3'. The reactions contained 10 μM template, 50 μM dATP, trace amounts of [α-^32^P] dATP, and 500 nM NrSPol, N300, or their mutants. After incubation for 30 min at 50°C, the products were separated on a 3 M urea-25% PAGE.

We identified a primase recognition sequence of NrSPol (5'-TTTGGTTA-3') in a previous study (Zhu et al., 2017), and a 23-nt DNA template containing the recognition site was used to evaluate the primase activities of the mutants in this study (Figure 2B; Supplementary Figure S3). HBD-inactivated mutants of NrSPol and N300 show very weak primase activities on the 23-nt DNA template (Figure 2B; Supplementary Figure S3). However, *de novo* synthesis products can be synthesized by HBD-inactivated mutants, especially by NrSFL-Y261A (Figures 4A,B). This led us to suspect that there could be other recognition sites, or the recognition sequence could be very flexible. We tested various DNA templates containing different sequence motifs and found that the 5'-GTGA-3' motif might be a primase recognition sequence that can be recognized by NrSPol (data not shown). To confirm the 5'-GTGA-3' motif as a novel primase recognition sequence, we replaced each of the 5'-GTGA-3' motif bases with all three possible alternative bases and tested the primer synthesis on these DNA templates. The results show that base substitution in the 5'-GTGA-3' motif affects the primer synthesis, especially the first three bases (5'-GTG-3'), where base substitutions severely impede the primer synthesis by N300 and NrSPol (Figure 5A; Supplementary Figure S4). The fourth base of the motif weakly affects the primer synthesis, and 5'-GTGA-3' and 5'-GTGG-3'motifs are two of the most efficient primase recognition sequences (Figure 5A; Supplementary Figure S4). Shortening the bases downstream of the recognition motif does not affect the primer synthesis (Supplementary Figure S5), further confirming the minimum primase recognition sequences. Because HBD is responsible for binding the primase recognition site (Boudet et al., 2019), the recognition of two distinct primase recognition sites by NrSPol indicates the flexible conformation of its HBD.

We next compared the primase activities of HBD-inactivated mutants of N300 and NrSPol on the DNA templates containing recognition sites 5'-GTGG-3' and 5'-TTTGGTTA-3'. The results showed that the newly identified primase recognition sequence (5'-GTGG-3') is more efficient than the previously identified sequence (5'-TTTGGTTA-3'; Figure 5B). The HBD-inactivated mutants of N300 and NrSPol, especially the Y261A mutants, also show weak primase activities on the template containing the 5'-GTGG-3' motif (Figure 5B), consistent with their activities for *de novo* DNA synthesis on M13 ssDNA (Figure 4A). In addition, we also tested the primase activities of other mutants of N300 and NrSPol on the template containing the newly identified recognition site (5'-GTGG-3'). Compared to the primase activities of N300 and NrSPol, the mutants show lower relative activities (Supplementary Figure S6), consistent with the results observed on the template containing the previously identified recognition site (Figure 2B; Supplementary Figure S3). It also shows that substitutions of the amino acid residues do not change the specificity of the primase recognition sequence.

### Flexibility in the Initiation Site of Primer Synthesis

**Figure 6:**
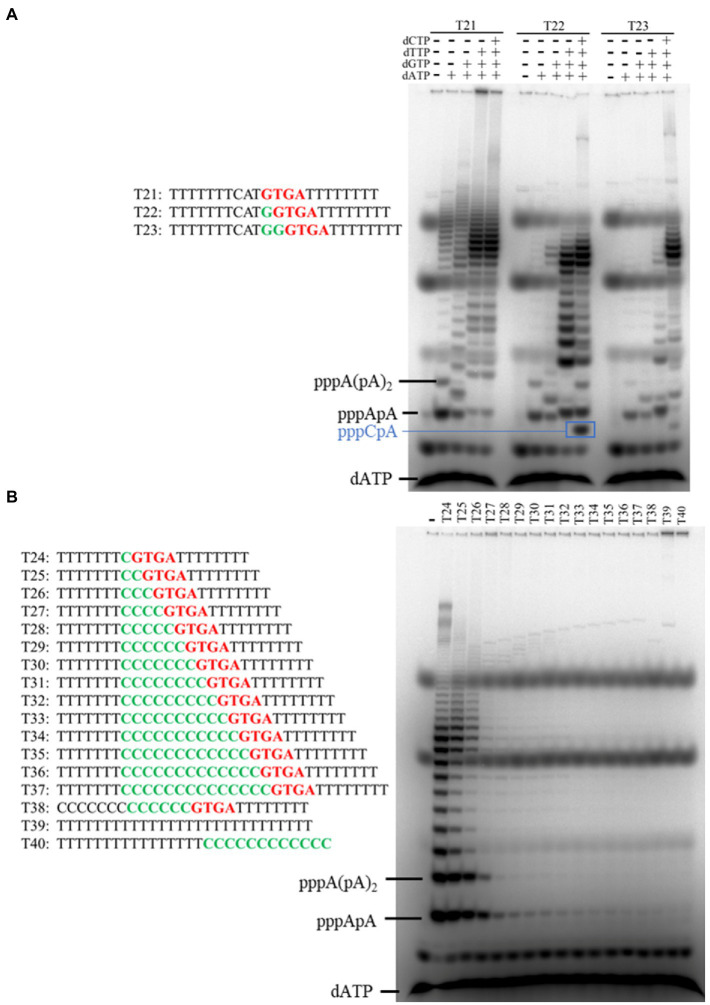
Identification of the initiation site of primer synthesis on the template containing primase recognition site 5'-GTGA-3'. **(A)** Primers were synthesized on different templates (T21–T23) in the presence of [α-^32^P] dATP and indicated dNTPs. **(B)** Primers were synthesized on different templates (T24–T40) in the presence of dATP and [α-^32^P] dATP. The products were separated on a 3 M urea-25% PAGE. Data in Figure **B** are representative of two independent experiments.

We have found that NrSPol initiates primer synthesis at the first base upstream of the recognition site and that it can also skip the first position to initiate primer synthesis (Zhu et al., 2017). In this study, we detailly investigated the initiation site of primer synthesis on the template containing the newly identified primase recognition site. Different dinucleotides and trinucleotides are produced when the first and second bases upstream of the 5'-GTGA-3' motif are replaced with all three possible alternative bases (Figure 5A), indicating that N300 also initiates primer synthesis at the first base upstream of the newly identified recognition site. Furthermore, different DNA templates containing the 5'-GTGA-3' recognition site were designed to investigate whether the enzyme can skip the first position of the initiation site. We tested primer synthesis on DNA templates T21, T22, and T23 in the presence of [α-^32^P] dATP and indicated dNTPs. Primers can be synthesized on the template T21 in the presence of only dATP and on the template T22 in the presence of dATP and dGTP, indicating that primer synthesis can be initiated at the fourth base upstream of the recognition site in principle (Figure 6A). On the template T23, N300 can initiate primer synthesis at the third base upstream of the recognition site, and the enzyme has a slight initiation synthesis activity even at the sixth base upstream of the recognition site (Figure 6A). To further confirm the slippage of the initiation site of primer synthesis, we designed a series of simple DNA templates containing the 5'-GTGA-3' recognition site and oligo (dT) sequences that were spaced by different lengths of oligo (dC) sequences, and tested the primer synthesis on these templates in the reaction mixtures containing only [α-^32^P] dATP and cold dATP. In principle, the poly (dA) primers can be effectively synthesized on these templates only if the enzyme can skip the oligo (dC) sequences. As expected, the amounts of synthesized primers decrease with increasing dC bases in the templates (Figure 6B). However, clear poly (dA) products can be synthesized on the template T26 (Figure 6B), indicating that N300 can skip three dC bases and initiate primer synthesis at the fourth base upstream of the recognition site. Compared to negative control templates T38, T39, and T40, trace amounts of poly (dA) products can still be synthesized on the template T32, in which oligo (dT) sequences are spaced by nine dC bases (Figure 6B). These results confirm that the enzyme has significant flexibility in the initiation site of primer synthesis. Because HBD is responsible for preparing initiation of primer synthesis (Boudet et al., 2019), the results also suggest that HBD has a highly flexible conformation.

### Y146 May Be Responsible for dNTP Selection as Preferred Substrate

**Figure 7:**
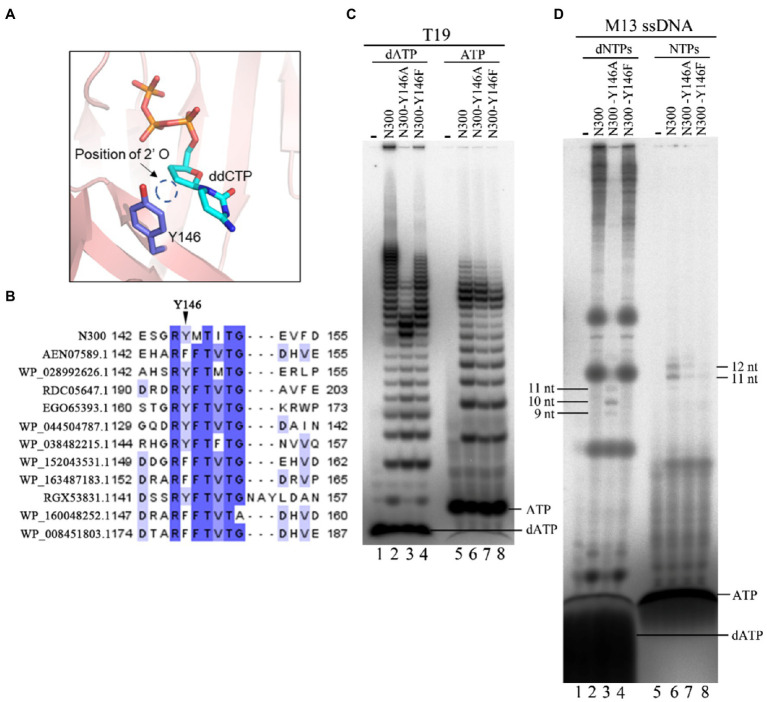
Effects of Y146 mutations in N300 on catalytic activity. **(A)** Detailed interaction between Y146 and the ribose of ddCTP. **(B)** Alignment of Y146 of N300 and equivalent positions to Y146 of N300 orthologs. **(C)** DNA primer synthesis (lanes 1–4) and RNA primer synthesis (lanes 5–8) on the template T19 [5'-(T)_14_TTGTGGTT-3']. Reactions were incubated for 30 min at 50°C in the presence of either dATP and [α-^32^P] dATP (lanes 1–4) or ATP and [α-^32^P] ATP (lanes 5–8). The products were separated on a 3 M urea-25% PAGE. **(D)** DNA synthesis (lanes 1–4) and RNA synthesis (lanes 5–8) on the M13 ssDNA. Reactions were incubated for 30 min at 50°C in the presence of either dNTPs and [α-^32^P] dATP (lanes 1–4) or NTPs and [α-^32^P] ATP (lanes 5–8). The products were separated on an 8 M urea-20% PAGE. Data in the figure are representative of at least two independent experiments.

In a previous study, Y146 was proposed to be involved in substrate discrimination (Guo et al., 2019). In this study, the N300 structure also showed that the 2' position of the ribose of incoming ddCTP is adjacent to Y146 (Figure 7A). Multiple sequence alignment of N300 with other PrimPol proteins showed that Y146 is not conserved but naturally substituted by a Phe residue in some PrimPol members (Figure 7B). To investigate the role of Y146, the residue was substituted with Ala and Phe. Y146A mutation in N300 dramatically reduces both primer extension and primase activities, while Y146F mutation in N300 only slightly affects the catalytic activity, suggesting that the side chain of Y146 strongly affects the catalytic activity of N300 (Figures 2A,B). Next, we further investigated the role of Y146 in the DNA or RNA primer synthesis on the template containing the newly identified recognition site. Consistently, N300-Y146A shows a weaker DNA primer synthesis activity than that of N300-Y146F which is weaker than that of N300 (Figure 7C, lanes 1–4). Interestingly, N300-Y146A shows a weaker RNA primer synthesis activity than that of N300, but its activity is stronger than that of N300-Y146F (Figure 7C, lanes 5–8). We next investigated the activities of N300, N300-Y146A, and N300-Y146F on the M13 ssDNA in the presence of dNTPs or NTPs. Both N300 and N300-Y146F can synthesize a large number of long DNA products, while N300-Y146A can only synthesize a trace amount of 9–11 nt DNA primers (Figure 7D, lanes 1–4). However, N300, N300-Y146A, and N300-Y146F can only synthesize a trace amount of 11–12 nt RNA primers in the presence of only NTPs, and in this case the activity of N300-Y146A is weaker than that of N300 but stronger than that of N300-Y146F (Figure 7D, lanes 5–8). These results indicate that Y146 may also play an important role in substrate discrimination in primer synthesis. Because Y146 is naturally replaced by the Phe residue at the equivalent position in many other PrimPols, in this case, the PrimPols may initiate DNA synthesis more exclusively with dNTPs.

## Discussion

It is traditionally accepted that DNA replication requires both DNA polymerase and primase in all domains of life. It is thus still unclear why *de novo* genome synthesis requires the two kinds of enzymes (Kuchta and Stengel, 2010; Zhu et al., 2017), even though the AEP superfamily proteins share a common ancestor with family B DNA polymerases, the replicative polymerases in archaea and eukaryotes (Iyer et al., 2005; Beck and Lipps, 2007). NrSPol is a PrimPol that belongs to the AEP superfamily. Although the enzyme is able to make long extension of DNA chain and is responsible for synthesizing the NrS-1 phage genome *de novo* (Zhu et al., 2017), it is not a conventional polymerase and its polymerization activity is much lower than that of the classical DNA polymerases. The HBD, a conserved domain in the AEP superfamily, is responsible for binding the primase recognition site and preparing initiation of primer synthesis (Boudet et al., 2019). In this study, we found that HBD-inactivating mutations in NrSPol dramatically decrease primase activity but enhance the DNA polymerization activity. It seems that the HBD actually hinders the DNA polymerization activity. Similarly, the Zn-finger domain of hPrimPol is critical for primer formation, while deletion of this domain conversely enhances the DNA polymerase activity (Mourón et al., 2013; Keen et al., 2014). These results also suggest that primase activity and DNA polymerase activity are not compatible in a single protein with the same catalytic domain, and this may be one reason for why two different enzymes, a DNA polymerase and a primase, are required for *de novo* DNA synthesis in most cases.

Notably, although the primer extension activity of N300 is weaker than that of its HBD-inactivated mutants, the processivity of N300 is higher than that of HBD-inactivated mutants of N300 (Figures 3A,B), indicating a weaker DNA binding by the HBD-inactivated N300 mutants. Interestingly, the processivity of HBD-inactivated mutants of NrSPol is higher than that of NrSPol (Figures 3A, 4A,B), indicating that the DNA-binding provided by the HBD does not benefit the processivity of DNA polymerization by the full-length enzyme. Compared to the N300, the full-length NrSPol has a C-terminus helicase domain that was found to have DNA binding affinity (Zhu et al., 2017). The enhanced processivity may be due to the DNA binding affinity of C-terminus helicase domains reducing the dissociation of the full-length mutants from the DNA template during DNA synthesis. PrimPol proteins are usually fused with helicase domains, but the roles of the helicase domains remain unclear (Sanchez et al., 2009; Kazlauskas et al., 2018; Chen et al., 2020). A recent study showed that this domain can enhance the DNA polymerase activity of NrSPol (Chen et al., 2020). In this study, our results indicate that the helicase domain can enhance the processivity of NrSPol due to its DNA binding affinity.

ORF904 requires dNTPs and ATP to initiate primer synthesis (Beck and Lipps, 2007). It has been demonstrated that ATP-induced HBD conformational changes of ORF904 are critical for ORF904 to bind to the primase recognition site and prepare initiation of primer synthesis (Boudet et al., 2019). However, NrSPol can initiate primer synthesis in the presence of only dNTPs, indicating that the HBD conformational changes of NrSPol may be induced by dNTP. In this study, we identified a novel primase recognition site that is stronger than the previously identified site. It is also possible that other primase recognition sites still exist. Owing to different nucleotide sequences of primase recognition sites, the HBD conformations of NrSPol that bind to the primase recognition sites should be different, showing the flexibility of the HBD conformation. Moreover, we also investigated the initiation site of primer synthesis and found that N300 mainly initiates primer synthesis at the first base upstream of the primase recognition site, while the enzyme can skip up to three bases and initiate primer synthesis at the fourth base upstream of the primase recognition site. This may also be due to the flexible conformation of HBD that binds to the primase recognition site for initiation of primer synthesis.

In this study, we found that NrSPol has two distinct primase recognition sites (5'-TTTGGTTA-3' and 5'-GTG-3'). Obviously, the number of 5'-GTG-3' sites is much more than that of 5'-TTTGGTTA-3' sites in the NrS-1 phage genome. The 5'-TTTGGTTA-3' sites may ensure NrSPol to initiate genome replication at a specific site, while NrSPol may require 5'-GTG-3' sites to restart replication after replication collapse. In addition, the ability to initiate primer synthesis outside of the recognition motif and the slippage of the initiating nucleotide suggest the flexibility of primer initiation synthesis of NrSPol, which facilitates NrSPol to restart replication after DNA damage.

NrS-1 phage lacks the zinc finger motif that is typically found in many other AEP family proteins such as ORF904, while it contains a loop (residues S108, P109, and S110) in the active site instead. The structure of N300 in complex with ddCTP showed that the loop is responsible for forming hydrogen bonds with the γ-phosphate of the incoming dNTP. Mutation analysis demonstrated that the loop, especially the residue S108 in the loop, is critical for the catalytic activity of NrSPol. As the zinc finger motif of ORF904 is also involved in incoming dNTP binding (Lipps et al., 2004), it seems that the SPS loop substitutes for the function of the zinc finger motif present in other PrimPols. In addition, it should be noted that both the zinc finger motif of ORF904 and the SPS loop of NrSPol are involved in 3'-site nucleotide binding, while the Zn-finger domain of hPrimPol is involved in 5'-site nucleotide binding.

Y146 is another critical residue for the primer extension activity and primase activity. Y146 is naturally substituted by Phe in some other PrimPols at the same position (Figure 7B), and Y146F mutation only slightly affects the primer extension activity and primase activity (Figures 2A,B). However, Y146A mutation significantly impairs the catalytic activity of N300, indicating that the side chain of Y146 plays an important role in the catalytic activity of N300. The N300 structure also shows that the 2' deoxyribose of ddCTP is close to the aromatic ring of Y146 (Figure 7A). This aromatic ring may affect the binding of N300 to the incoming dNTP, as it has been found that Y146A mutation nearly abolished the binding capability of N300 with dNTP (Guo et al., 2019). We found that although the DNA primer synthesis activity of N300-Y146A is much weaker than that of N300-Y146F, its RNA primer synthesis activity is stronger than that of N300-Y146F (Figures 7C,D). That may be because Y146 is involved in recognizing and stabilizing the incoming dNTP. When Y146 is substituted by Ala, the catalytic activity of N300 is decreased dramatically. Correspondingly, the efficiency of discrimination against NTP is decreased by Y146A mutation.

In summary, in this study, we identified key residues that are important for the catalytic activity of NrSPol. Remarkably, we found that HBD-inactivating mutations conversely enhance DNA polymerization activity, indicating the conflict between DNA polymerase and primase activities within one protein. Moreover, the flexible primase recognition and initiation sites of NrSPol suggest its flexible HBD conformation.

## Data Availability Statement

The datasets presented in this study can be found in online repositories. The names of the repository/repositories and accession number(s) can be found in the article/Supplementary Material.

## Author Contributions

Experiments were designed by BZ, FH, and LW. Biochemical experiments were conducted by FH with assistance from XL. X-ray crystallography data were collected by LW, CY, and PS. The manuscript was written by FH, LW, and BZ. All authors contributed to the article and approved the submitted version.

## Funding

This project is funded by the National Natural Science Foundation of China (grants 31870165 to BZ and 31900032 to FH) and Shenzhen Science and Technology Innovation Fund (grant JCYJ20210324115811032 to BZ). Funding for open access charge: National Natural Science Foundation of China.

## Conflict of Interest

The authors declare that the research was conducted in the absence of any commercial or financial relationships that could be construed as a potential conflict of interest.

## Publisher’s Note

All claims expressed in this article are solely those of the authors and do not necessarily represent those of their affiliated organizations, or those of the publisher, the editors and the reviewers. Any product that may be evaluated in this article, or claim that may be made by its manufacturer, is not guaranteed or endorsed by the publisher.
