# Corrigendum: Molecular Dissection of the Primase and Polymerase Activities of Deep-Sea Phage NrS-1 Primase-Polymerase

**DOI:** 10.3389/fmicb.2021.838050

**Published:** 2022-01-11

**Authors:** Fengtao Huang, Xueling Lu, Chunxiao Yu, Piotr Sliz, Longfei Wang, Bin Zhu

**Affiliations:** ^1^Key Laboratory of Molecular Biophysics, The Ministry of Education, College of Life Science and Technology and Shenzhen College, Huazhong University of Science and Technology, Wuhan, China; ^2^Department of Biological Chemistry and Molecular Pharmacology, Harvard Medical School, Boston, MA, United States; ^3^Program in Cellular and Molecular Medicine, Boston Children's Hospital, Boston, MA, United States; ^4^School of Pharmaceutical Sciences, Wuhan University, Wuhan, China

**Keywords:** DNA polymerase, primase, DNA replication, *de novo* synthesis, phage

In the published article, there was an error regarding the affiliations for Longfei Wang. As well as having affiliations 2 and 3, they should also have affiliation 4.

The authors apologize for this error and state that this does not change the scientific conclusions of the article in any way. The original article has been updated.

## Publisher's Note

All claims expressed in this article are solely those of the authors and do not necessarily represent those of their affiliated organizations, or those of the publisher, the editors and the reviewers. Any product that may be evaluated in this article, or claim that may be made by its manufacturer, is not guaranteed or endorsed by the publisher.

