# Supplementary material for: Molecular Dissection of the Primase and Polymerase Activities of Deep-Sea Phage NrS-1 Primase-Polymerase
Source: Front Microbiol. 2021 Dec 17;12:766612. doi: 10.3389/fmicb.2021.766612 (PMC8718748; doi:10.3389/fmicb.2021.766612)
Supplement: Supplementary file 1 [file Data_Sheet_1.PDF]

Supplementary Materials

for

Molecular dissection of the primase and polymerase activities of deep-sea phage NrS-1 primase-polymerase

Fengtao Huang[1]*, Xueling Lu[1], Chunxiao Yu[2], Piotr Sliz[2], Longfei Wang[2,3]*, Bin Zhu[1]*

[1]Key Laboratory of Molecular Biophysics, the Ministry of Education, College of Life Science and Technology and Shenzhen College, Huazhong University of Science and Technology, Wuhan, Hubei 430074, China;

[2]Department of Biological Chemistry and Molecular Pharmacology, Harvard Medical School, and Program in Cellular and Molecular Medicine, Boston Children's Hospital, Boston, MA 02115, USA

[3]School of Pharmaceutical Sciences, Wuhan University, Wuhan, 430071, China

*To whom correspondence should be addressed. Email: huang_fengtao@126.com; wanglf@whu.edu.cn; bin_zhu@hust.edu.cn

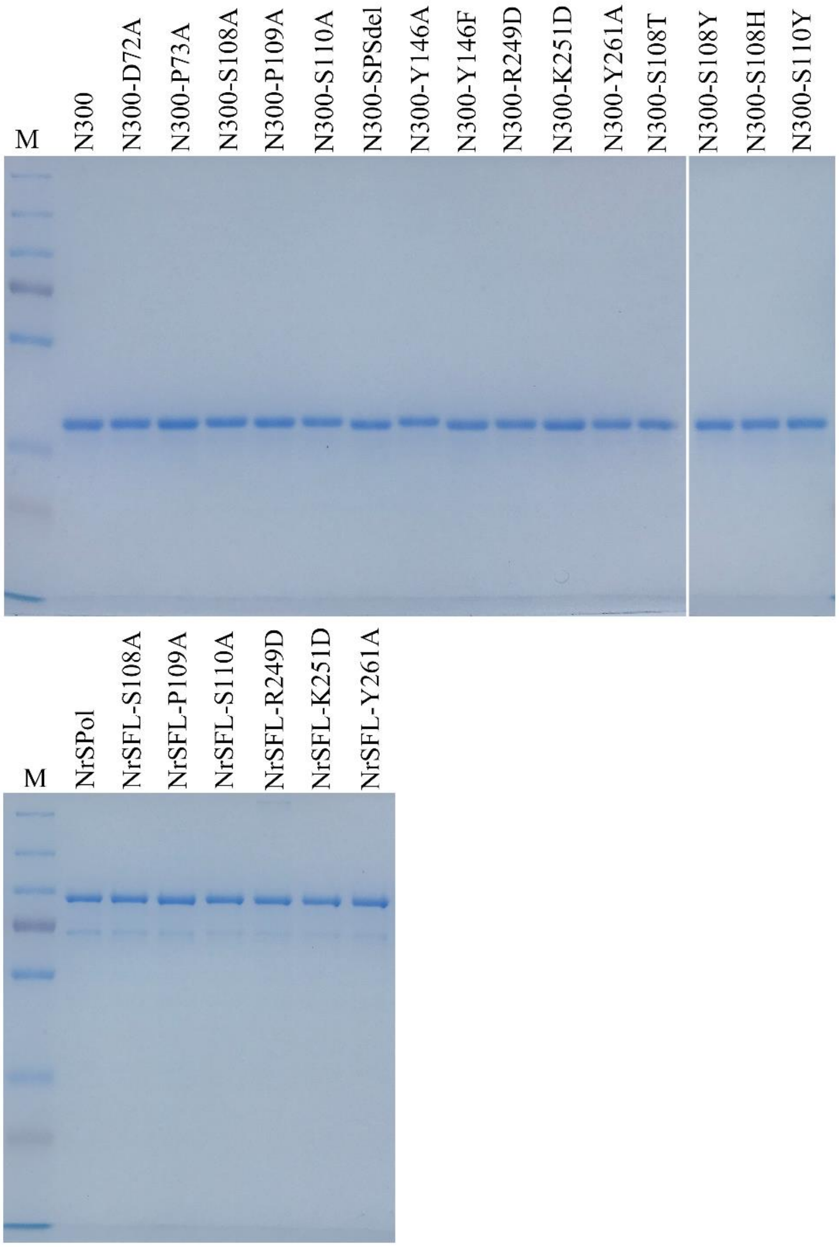

Figure S1. SDS-PAGE analysis of the purified proteins in this work.

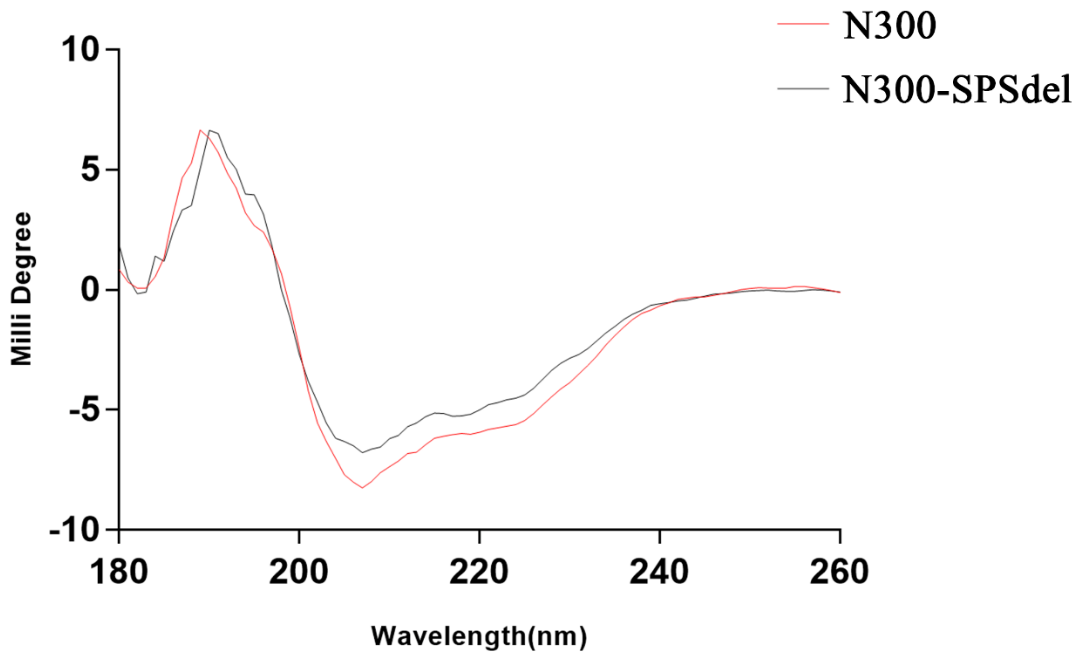

Figure S2. Circular-dichroism spectra of N300 and the mutant N300-SPSdel.

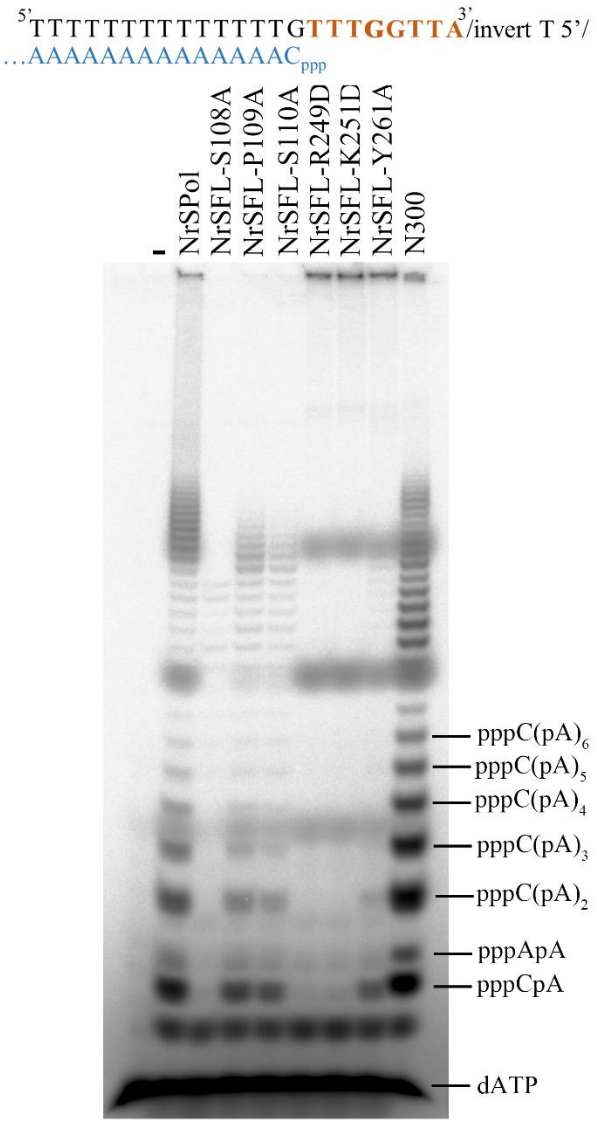

Figure S3. The primase activities of NrSPol and its mutants on the designed 23-nt DNA template. Related to Figure 2B. The reaction mixtures (10 μl) contained 10 μM of the 23-nt DNA template, 50 μM dCTP, 50 μM dATP, trace amounts of [α-^{32}P] dATP, and 0.5 μM NrSPol, its mutants, or N300. Reactions were incubated for 30 min at 50°C and then were stopped by adding 5 μl of 95% formamide dye containing 15 mM EDTA. Samples were boiled for 3 min at 90°C and analyzed by 25% PAGE containing 3 M urea.

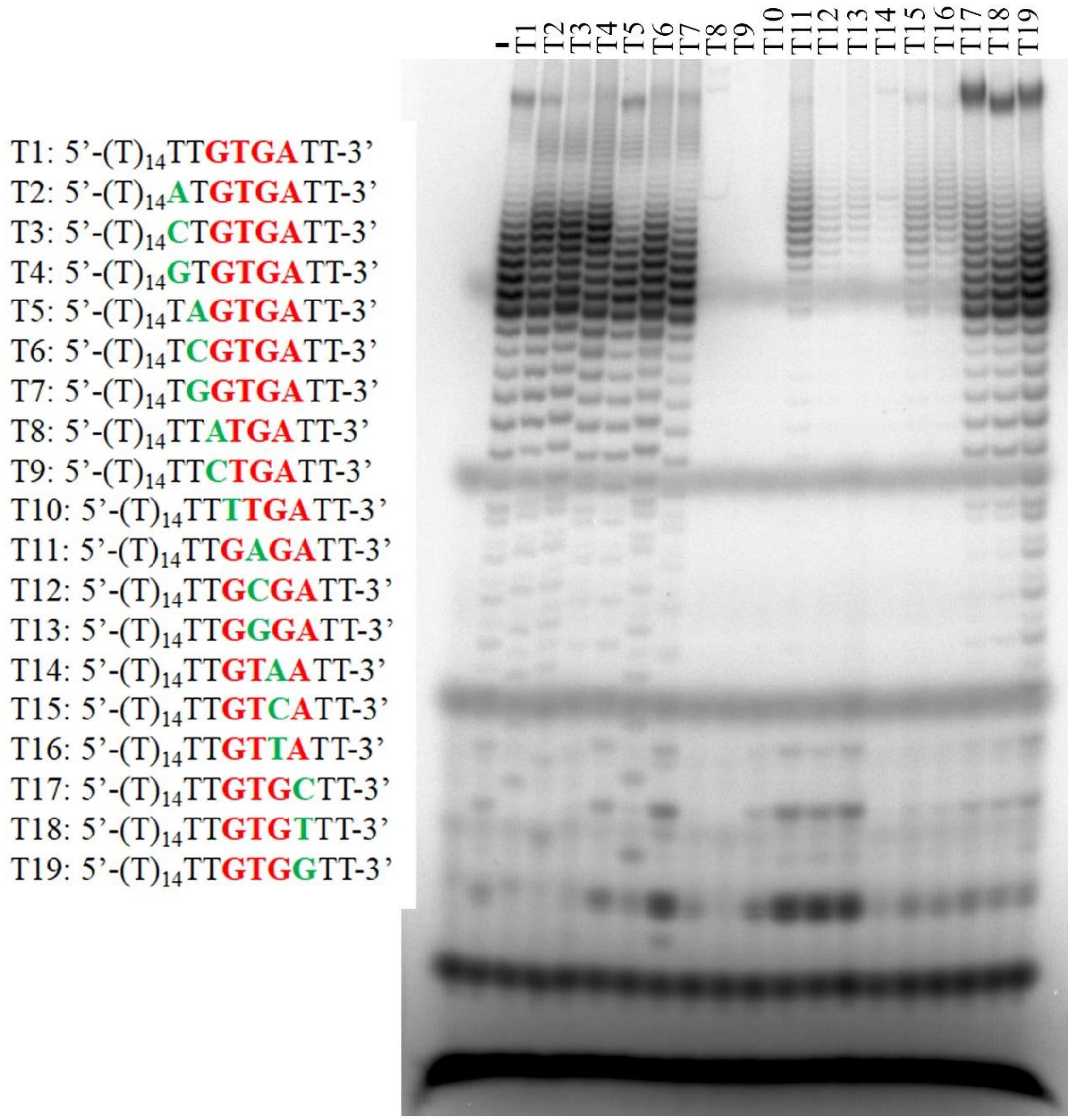

Figure S4. Dissection of the DNA recognition sequence of NrSPol. Related to Figure 5A. The reaction mixtures (10 µl) contained 0.5 µM NrSPol, 10 µM different oligodeoxynucleotide templates, 50 µM dNTPs, and trace amounts of [α-^{32}P] dATP. Reactions were incubated for 30 min at 50°C and then were stopped by adding 5 µl of 95% formamide dye containing 15 mM EDTA. Samples were boiled for 3 min at 90°C and analyzed by 25% PAGE containing 3 M urea.

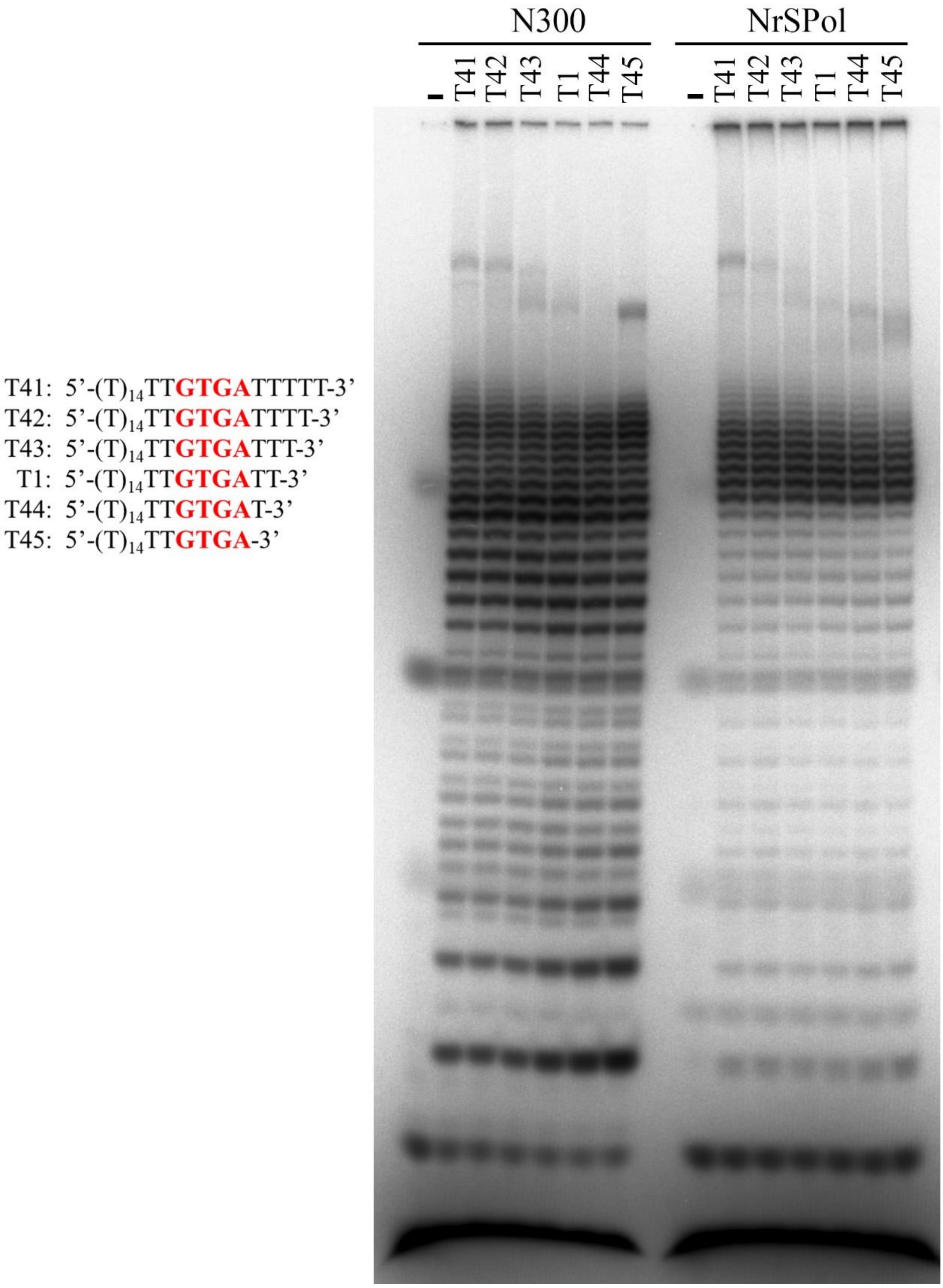

Figure S5. Primer synthesis by N300 and NrSPol on the DNA templates with shortened 3' end. Related to Figure 5A. The reaction mixtures (10 μl) contained 10 μM different oligodeoxynucleotide templates, 50 μM dNTPs, trace amounts of [α-^{32}P] dATP, and 0.5 μM NrSPol or N300. Reactions were incubated for 30 min at 50°C and then were stopped by adding 5 μl of 95% formamide dye containing 15 mM EDTA. Samples were boiled for 3 min at 90°C

and analyzed by 25% PAGE containing 3 M urea.

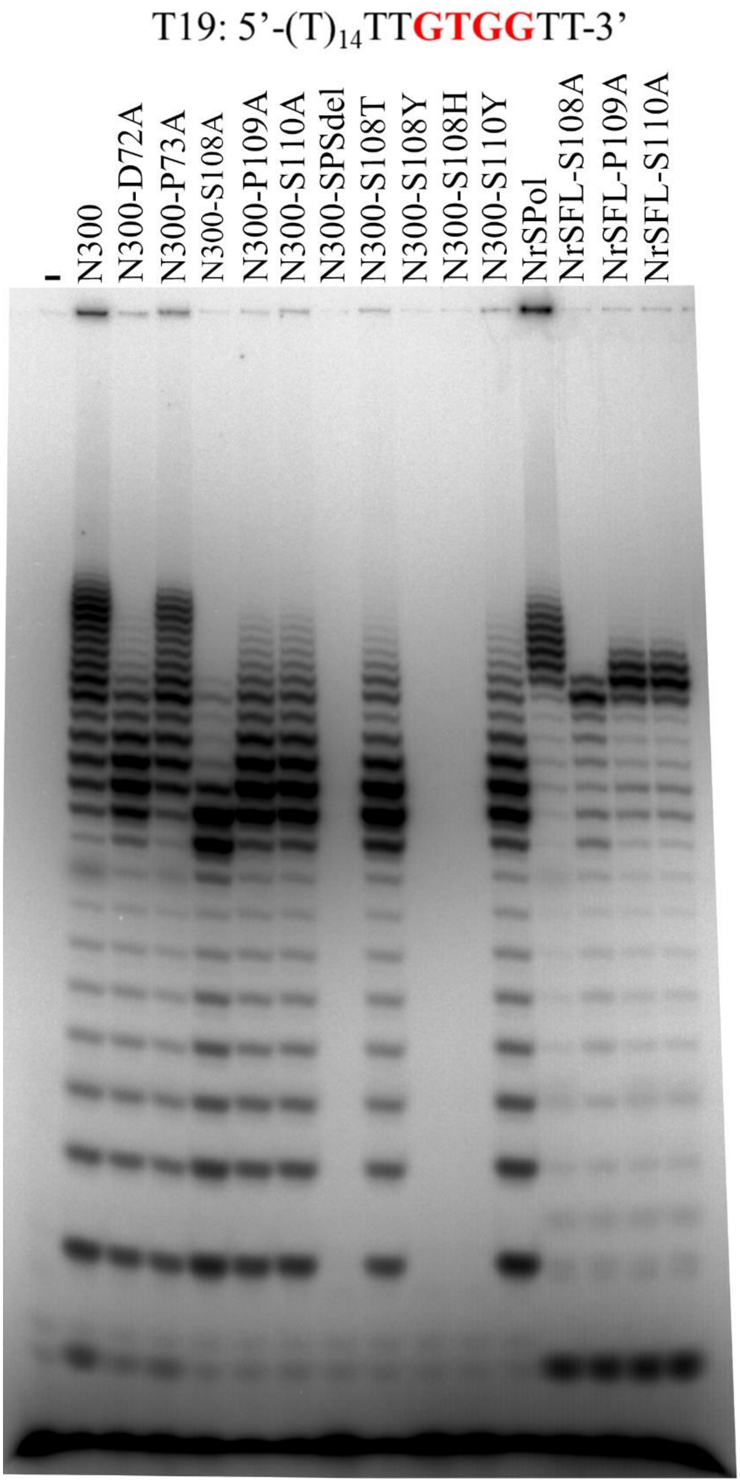

Figure S6. Comparison of primase activities of NrSPol, N300, and their mutants on template T19 containing the primase recognition site 5'-GTGG-3'. Related to Figure 5B. The reaction mixtures (10 μl) contained 10 μM template T19, 50 μM dATP, trace amounts of [α-^{32}P] dATP, and 0.5 μM NrSPol, N300, or their mutants. Reactions were incubated for 30 min

at 50°C and then were stopped by adding 5 µl of 95% formamide dye containing 15 mM EDTA. Samples were boiled for 3 min at 90°C and analyzed by 25% PAGE containing 3 M urea.

Table S1. Data collection and refinement statistics.

	**N300 PPi Mg^{2+}**	**SeMet N300 ddCTP Ca^{2+}**
**Wavelength (Ã)**	0.97919	0.97920
**Resolution range (Ã)**	51.34 - 1.86 (1.926 - 1.86)	51.1 - 2.24 (2.32 - 2.24)
**Space group**	C 1 2 1	P 21 21 21
**Unit cell**	119.34 56.903 48.564 90 93.6 90	56.452 71.58 72.962 90 90 90
**Total reflections**	93633 (9408)	56088 (5516)
**Unique reflections**	26979 (2708)	14442 (1419)
**Multiplicity**	3.4 (3.5)	3.9 (3.9)
**Completeness (%)**	98.29 (98.50)	97.90 (98.39)
**Mean I/sigma(I)**	12.80 (1.19)	11.39 (1.40)
**Wilson B-factor**	38.65	44.39
**R-merge**	0.09634 (1.175)	0.1572 (1.35)
**R-meas**	0.1157	0.1848
**CC1/2**	0.385 (0.622)	0.236 (0.615)
**CC***	0.746 (0.876)	0.618 (0.873)
**Reflections used for R-free**		
**R-work**	0.2044 (0.3703)	0.2302 (0.3743)
**R-free**	0.2193 (0.3914)	0.2889 (0.4618)
**Number of non-hydrogen atoms**	2249	2197
**macromolecules**	2123	2127
**ligands**	18	30

**water**	108	40
**Protein residues**	275	276
**RMS(bonds)**	0.006	0.003
**RMS(angles)**	1.08	0.95
**Ramachandran favored (%)**	97	95
**Ramachandran allowed (%)**		
**Ramachandran outliers (%)**	1.1	1.1
**Clashscore**	7.92	5.03
**Average B-factor**	48.40	50.60
**macromolecules**	48.10	50.60
**ligands**	67.70	54.10
**solvent**	50.00	49.80
